# Electronegativity and doping in Si_1-*x*_Ge_*x*_ alloys

**DOI:** 10.1038/s41598-020-64403-8

**Published:** 2020-05-04

**Authors:** Stavros-Richard G. Christopoulos, Navaratnarajah Kuganathan, Alexander Chroneos

**Affiliations:** 1 Faculty of Engineering, Environment and Computing, Coventry University, Priory Street, Coventry, CV1 5FB United Kingdom; 2 Department of Materials, Imperial College London, London, SW7 2AZ United Kingdom

**Keywords:** Density functional theory, Electronic devices

## Abstract

Silicon germanium alloys are technologically important in microelectronics but also they are an important paradigm and model system to study the intricacies of the defect processes on random alloys. The key in semiconductors is that dopants and defects can tune their electronic properties and although their impact is well established in elemental semiconductors such as silicon they are not well characterized in random semiconductor alloys such as silicon germanium. In particular the impact of electronegativity of the local environment on the electronic properties of the dopant atom needs to be clarified. Here we employ density functional theory in conjunction with special quasirandom structures model to show that the Bader charge of the dopant atoms is strongly dependent upon the nearest neighbor environment. This in turn implies that the dopants will behave differently is silicon-rich and germanium-rich regions of the silicon germanium alloy.

## Introduction

The evolution in dielectrics and the introduction of high dielectric constant (high-*k*) dielectrics has allowed the replacement of silicon (Si) with better materials such as germanium (Ge) and silicon germanium (Si_1−*x*_Ge_*x*_)^1–10^. Random alloys such as Si_1-*x*_Ge_*x*_ are different to elemental semiconductors as two atom species can occupy one lattice site. The consequence is that there is inhomogeneity of the local environments as there will be Si-rich and Ge-rich regions that will in turn influence the energetics of defect processes^3,6,11,12^. From a thermodynamic viewpoint, Saltas *et al*^11^. investigated self-diffusion in Si_1-*x*_Ge_*x*_ with respect to temperature and Ge concentration using the *cBΩ* thermodynamic model^13–16^. Saltas *et al*.^11^ identified significant deviations from linearity of the activation energies with respect to compositions and attributed this non-linear behaviour to the bulk properties of Si and Ge.

From a density functional theory (DFT) perspective modelling random alloys can be computationally intensive (or even intractable depending on the issue under investigation) as it necessitates a high number of calculations and very large supercells (>10^3^ atoms)^17^. As it has been previously discussed the special quasirandom structures (SQS) approach^17^ can lead to manageable supercells while mimicking the statistics of random alloys^18–23^.

The present study aims to use SQS in synergy with DFT to study the effect of electronegativity on doping in Si_1-*x*_Ge_*x*_ alloys (x = 0.125, 0.25, 0.375, 0.5, 0.625, 0.75, 0.875). The most significant *n*-type dopants (N, P, As and Sb) and *p*-type dopants (B, Al, Ga and In) are considered as they exhibit a range of electronegativities. From a fundamental viewpoint the association of composition, electronegativity and doping is important to understand and improve the electronic properties of alloy semiconductors.

## Results and Discussion

### Structure of Si_1-*x*_Ge_*x*_ alloys

The reasoning and efficacy of SQS to describe Si_1-*x*_Ge_*x*_ alloys and other related random alloys (for example Sn_1-*x*_Ge_*x*_ and Si_1-*x-y*_Ge_*x*_Sn_y_) has been discussed extensively in previous work and therefore here we will only briefly discuss this approach for completeness^24,25^. Typical DFT calculations of perfectly ordered structures require the formation of a supercell that is expanded throughout space by the use of periodic boundary conditions. For disordered random alloys the analogous approach is not practically feasible as it requires the construction of very large supercells (>10^3^ atoms)^17^ with the atoms being randomly positioned at the lattice sites. The advantage of the SQS method is that it efficiently mimics the statistics of random alloys with small supercells (for example 16–32 atoms in Si_1-*x*_Ge_*x*_)^25^ and this allows the practical application of DFT in materials were many defect calculations are needed^18–23^. A key advantage of the SQS approach is that the atomistic nature is maintained and this leads to a distribution of distinct local environments that exist in real random alloys.

### Local environment and dopant electronegativity

Previous theoretical and experimental studies have established that there is a non-linear dependence of defect processes (for example binding energies of *E*-centres or activation energies of self- and dopant diffusion) with respect to composition^26–31^. Not only relaxation and thermodynamics impact the defect processes but also the electronic properties of the defects (for example charge transfer) can influence the properties of Si_1-*x*_Ge_*x*_ and in that respect the local environment is anticipated to play a role on the formation of *n*-type and *p*-type doped areas in Si_1-*x*_Ge_*x*_. What is the impact of electronegativity of dopants and the local environment in Si_1-*x*_Ge_*x*_ alloys?

Here we considered full geometry optimizations (positions and cell) of *n*-type dopants (N, P, As and Sb) and *p*- type dopants (B, Al, Ga and In) in Si_1-x_Ge_x_ configurations (x = 0.125, 0250, 0.375, 0.500, 0.625, 0.750 and 0.875) to examine the local coordination of the dopants formed with the nearest neighbour atoms (Si and Ge). The simulation technique permitted us to determine the Bader charge^32,33^ on each dopant and the nearest neighbour atoms attached to the dopants in the relaxed configurations. The Bader charge approximation enabled us to discuss the electronegativity trend of the dopants present in Si_1-x_Ge_x_ alloys. In the Bader charge analysis, electronic charges on individual atoms in the lattice are calculated based on the partitioning method as implemented by Bader^34^. Zero flux surfaces are used to divide atoms and partition the charge density^32,33^. A zero flux surface of the gradients of the electron density is expressed by the following equation as discussed by Yu *et al*^35^.

1 $$\nabla \rho (\overrightarrow{r})\cdot \hat{n}=0$$

Where $\rho (\overrightarrow{r})$ is the electron density, and the $\hat{n}$ is the unit vector perpendicular to the dividing surface at any surface point $\overrightarrow{r}$. Partition of charges are based on the continuum probability density of trajectories $[P(\overrightarrow{r},t)]\,$ as defined by the following equation:

2 $$\overrightarrow{j}(\overrightarrow{r},t)=P(\overrightarrow{r},t)\nabla \rho (\overrightarrow{r})$$

Where $\overrightarrow{j}$ ($\overrightarrow{r}$,t) is the probability flux at any point and time.

**Figure 1:**
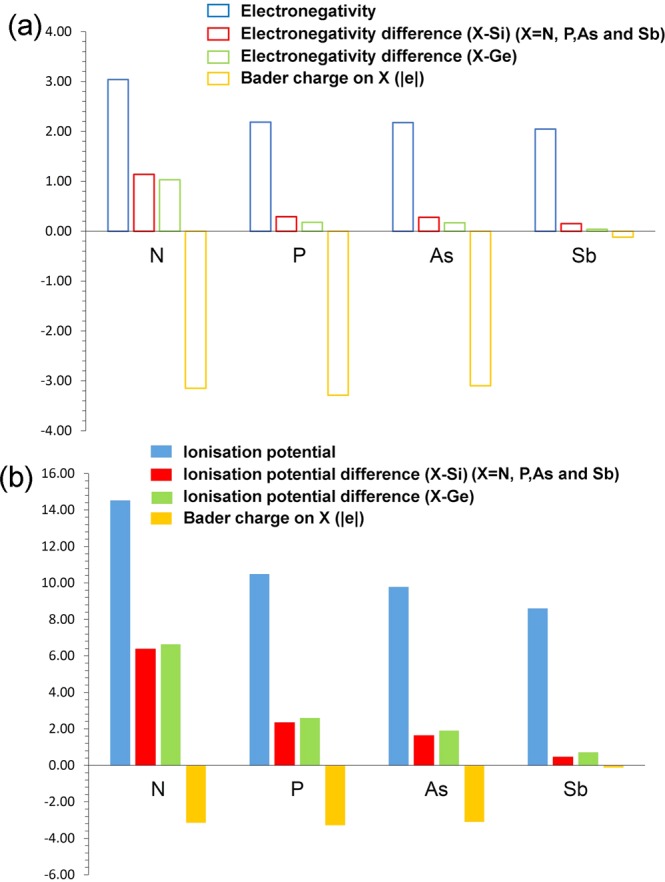
(**a**) Electronegativities of *n-*type dopants^34^, differences in the electronegativities between the dopants and Si (Ge) and the Bader charges on the dopants and (**b**) ionisation potentials of *n*-type dopants, differences in the ionisation potentials^35^ between the dopants and Si (Ge) and the Bader charges on the dopants.

**Figure 2:**
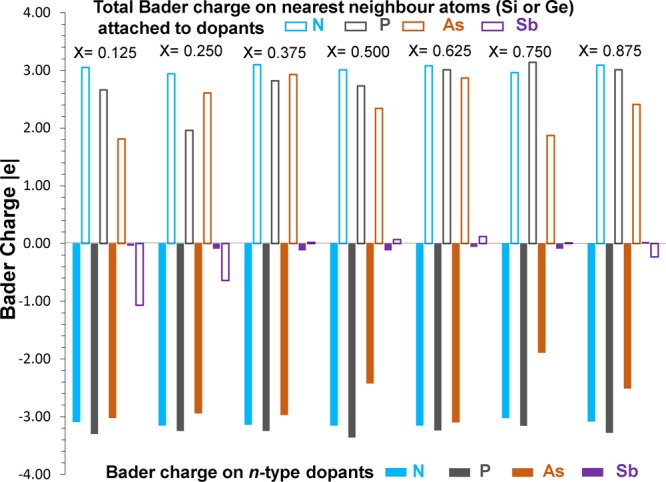
Calculated Bader charges on *n-*type dopants and sum of the Bader charges of Si and Ge atoms bonded to dopants.

**Figure 3:**
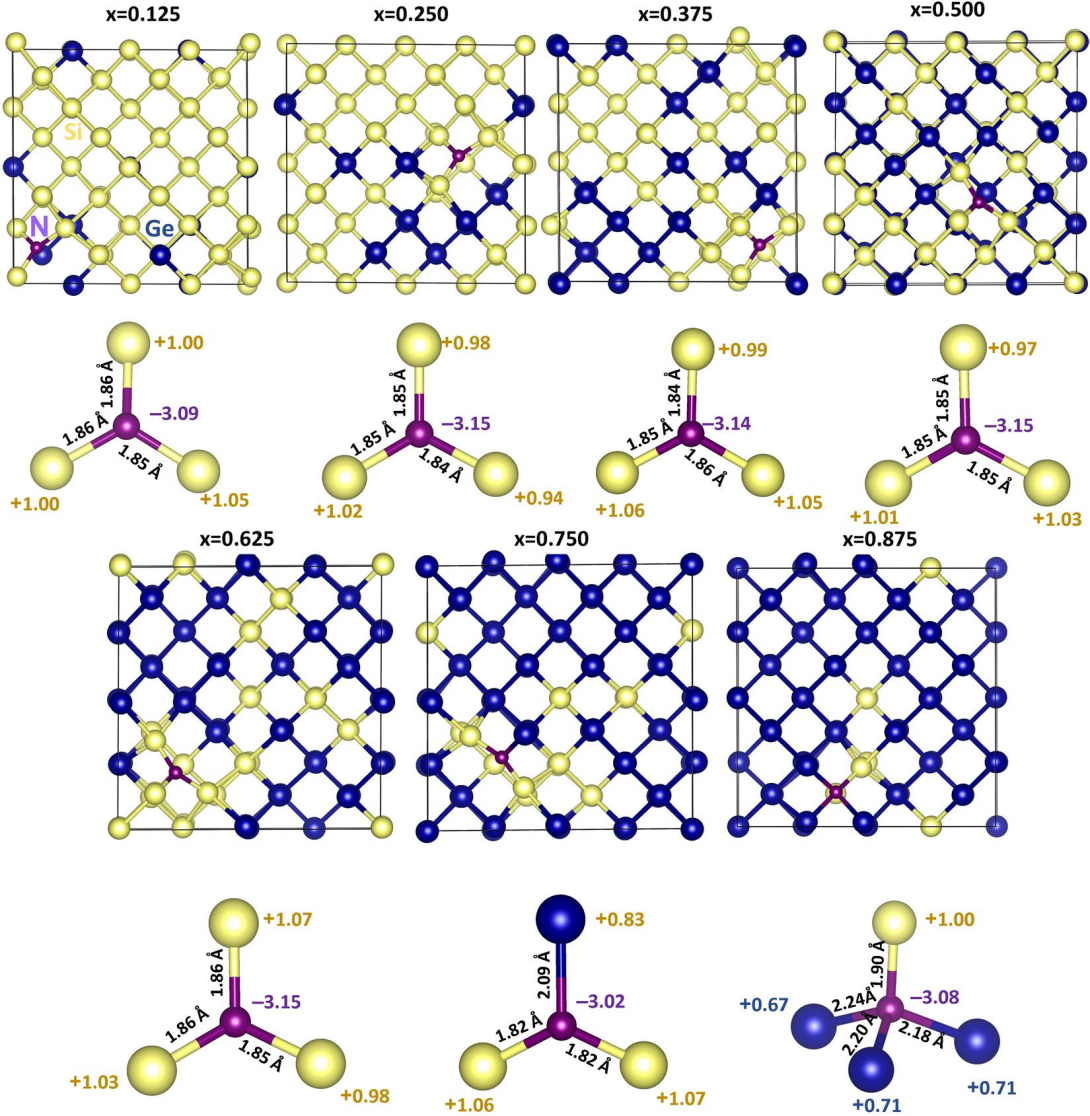
Optimised structures of seven different nitrogen substitutional defect configurations in Si_1−x_Ge_x_ alloys. Bader charges on the N and its nearest neighbour atoms and bond distances (N-Si and N-Ge) are also shown.

**Figure 4:**
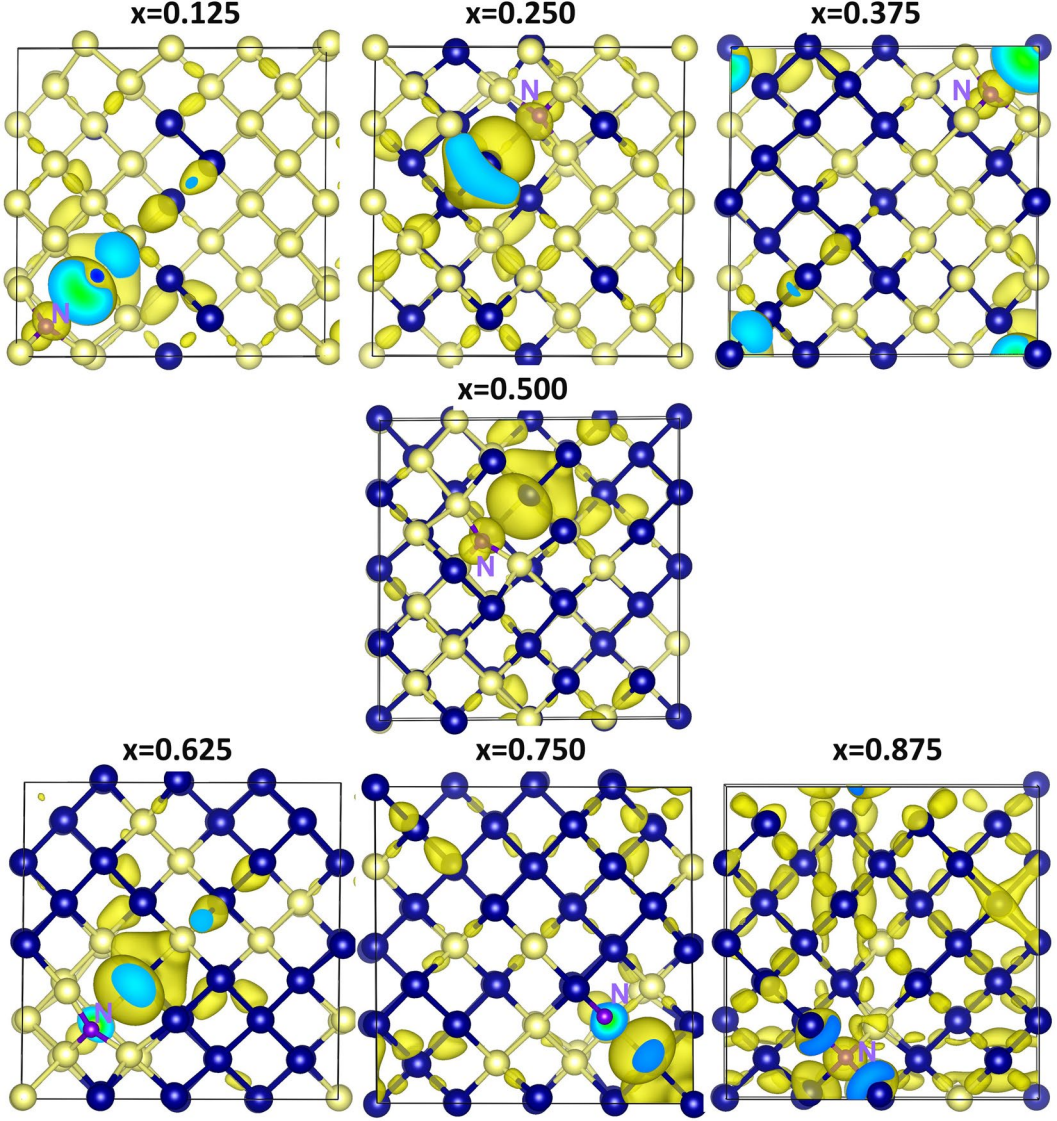
Surface of the constant charge density showing the interaction of nitrogen in each of the seven different configurations in Si_1−x_Ge_x_ alloys.

First we discuss the correlation observed between the electronegativity (or ionization potential) of *n*-type dopants and their Bader charges. Figure 1 report the electronegativity values^36^ of dopants, the electronegativity difference between dopants and Si (or Ge) and calculated Bader charges on the dopants. Electronegativities of N, Si and Ge are reported being 3.04, 1.90 and 2.01 respectively^36^. As nitrogen has higher electronegativity than that of Si or Ge, electronegativity difference (N-Si = 1.14 or N-Ge = 1.03) is positive. This reflects in the negative Bader charge on N (−3.15). Here we discuss the results for one of the seven configurations. Figure 2 reports the Bader charge on each dopants in seven different configurations and the sum of the Bader charges of the nearest neighbor atoms (Si or Ge). It is clear that in all seven configurations N gains ~3.00 electrons from Si and Ge to complete its outer shell. Total Bader charge of Si and Ge attached to N is ~+3.00 confirming that those three electrons are transferred from Si and Ge. Figure 3 shows the relaxed structures of all seven configurations, Bader charges and bond distances (N-Si and N-Ge). In all seven configurations, the Bader charge on the Si is ~+1.00. There are two different coordination environments observed. In the first five configurations (x = 0.125–0.625), N forms a trigonal planar structure with three nearest neighbor Si atoms. Each Si atom transfers ~1.00 electron to N to form a N^3-^ stable electronic configuration. The N-Si bond distance in all configurations are ~1.85 Å showing the strong bonding formed between the N and the Si. In the sixth configuration (x = 0.750), a trigonal planar structure is formed two Si and one Ge atoms with N. In this case, each Si atom loses ~one electron and Ge atom loses 0.83 electrons. Slight reduction in the charge transfer by Ge is due to the lower electronegativity difference between N and Ge compared to that between N and Si^36^. This reflects in the longer bond distance of N-Ge than N-Si. In the last configuration (x = 0.875), N forms a distorted tetrahedral coordination with three Ge atoms and one Si atom. All three Ge atoms transfer ~0.70 electrons and one Si atom loses 1.00 electron leaving ~3.00 electrons on the N atom. Long N-Ge distances (~2.20 Å) confirms the weak bonding and reduction in the charge transfer. Figure 4 shows constant charge density plots associated with the dopants.

Electronegativity values of P and As are 2.19 and 2.18 respectively and these values are slightly larger than the values of Si and Ge (refer to Fig. 2). Electronegativity difference between P (or As) and Si (or Ge) is still positive but smaller than that observed between N and Si (or Ge). However, both P and As gain ~3.00 electrons. Relaxed configurations of P together with the bond distances and Bader charges are reported in Figure S1 in the supplementary information. Corresponding charge density plots are shown in Figure S2. In all seven configurations, P forms a tetrahedral coordination. Bader charges on Si or Ge varies from +0.60 to +0.81. Bond distances are longer compared to those observed in N•Si_1-x_Ge_x_. This is due to the lower electronegativity and larger atomic radius of P than that of N.

The Bader charges on the As in each configuration is negative and their magnitude vary from −1.89 to −3.10 (refer to Figs. 2 and S3). The negative charges on the As atoms are due to lose of electrons from Si and Ge as evidenced by the positive Bader charges (from +0.16 to +0.78). The formation of tetrahedral unit is observed in all cases with the longer bond distance as expected due to the larger atomic radius of As compared to that of N and P. Charge density plots are shown in Figure S4 in the supplementary information.

Electronegativity difference between Sb and Si (or Ge) is very small (refer to Fig. 1). This is reflected in the Bader charge on Sb. A very small amount of negative or positive charge is observed on the Sb atom in all cases (refer to Figs. 2 and S5). Total Bader charges on the nearest neighbor atoms are also small. Long Sb-Si or Sb-Ge bond distances are observed due to the large atomic radius of Sb and small electronegativity difference. Charge density plots are shown in Figure S6 in the supplementary information.

Charge transfer between dopants and Si (or Ge) can be explained in terms of ionization potentials. Figure 1 b shows the ionization potentials^37^ of dopants and Si and Ge. The largest ionization potential is observed for N. Ionization potential difference between N and Si (or Ge) is observed. This implies that the formation of positive charge on N is highly unlikely or gaining of electrons to form negative charge is highly likely. Ionization potential decreases from N to Sb meaning that the formation of positive charge become slightly favourable or gaining of electrons become less favourable.

**Figure 5:**
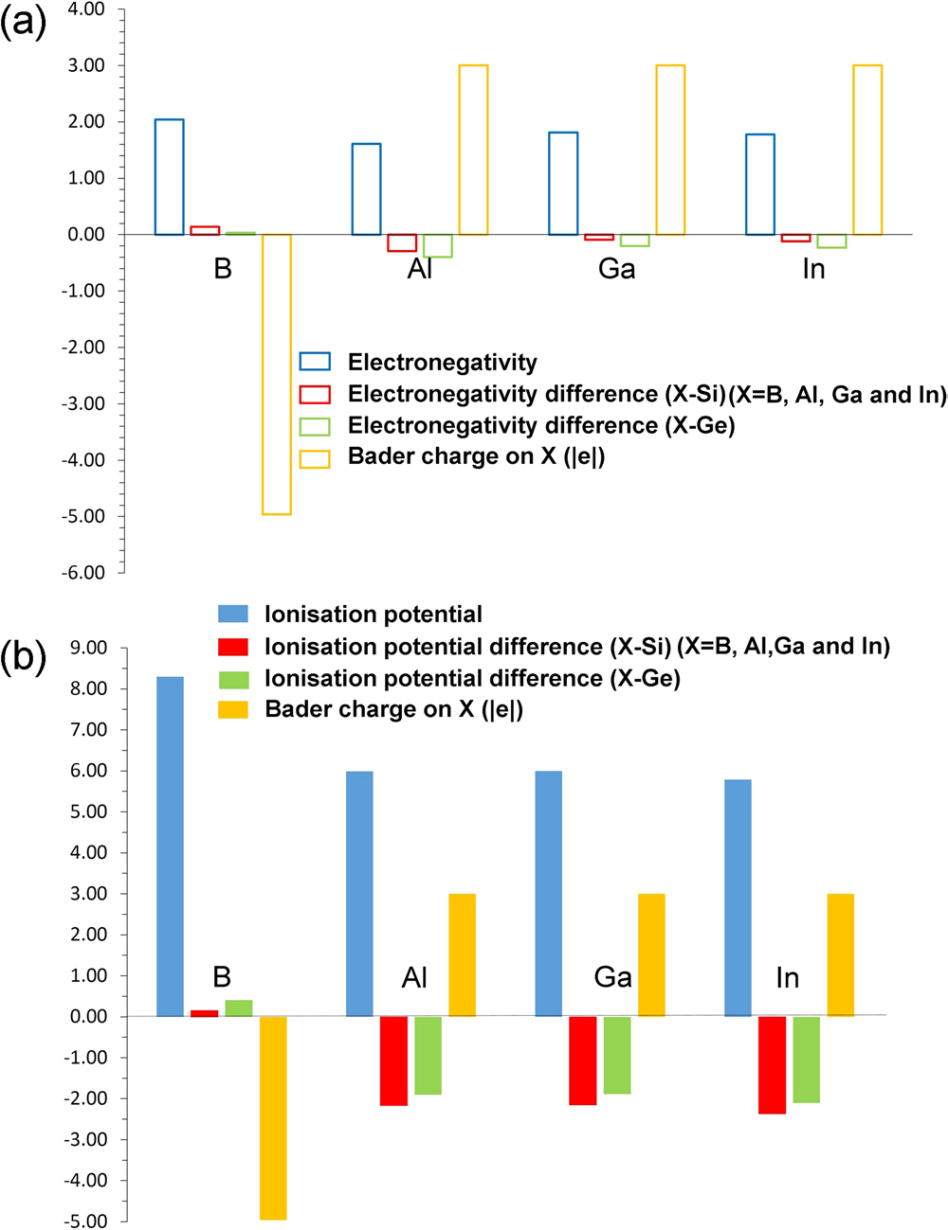
(**a**) Electronegativities of *p-*type dopants^34^, differences in the electronegativities between the dopants and Si (Ge) and the Bader charges on the dopants and (**b**) ionisation potentials of *p*-type dopants, differences in the ionisation potentials^35^ between the dopants and Si (Ge) and the Bader charges on the dopants.

**Figure 6:**
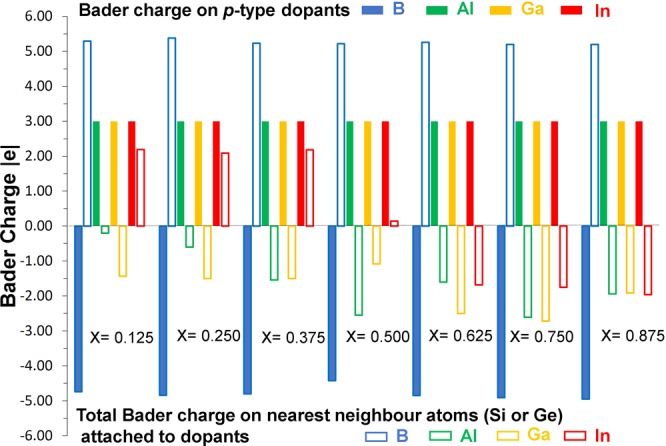
Calculated Bader charges on *p-*type dopants and sum of the Bader charges of Si and Ge atoms bonded to dopants.

**Figure 7:**
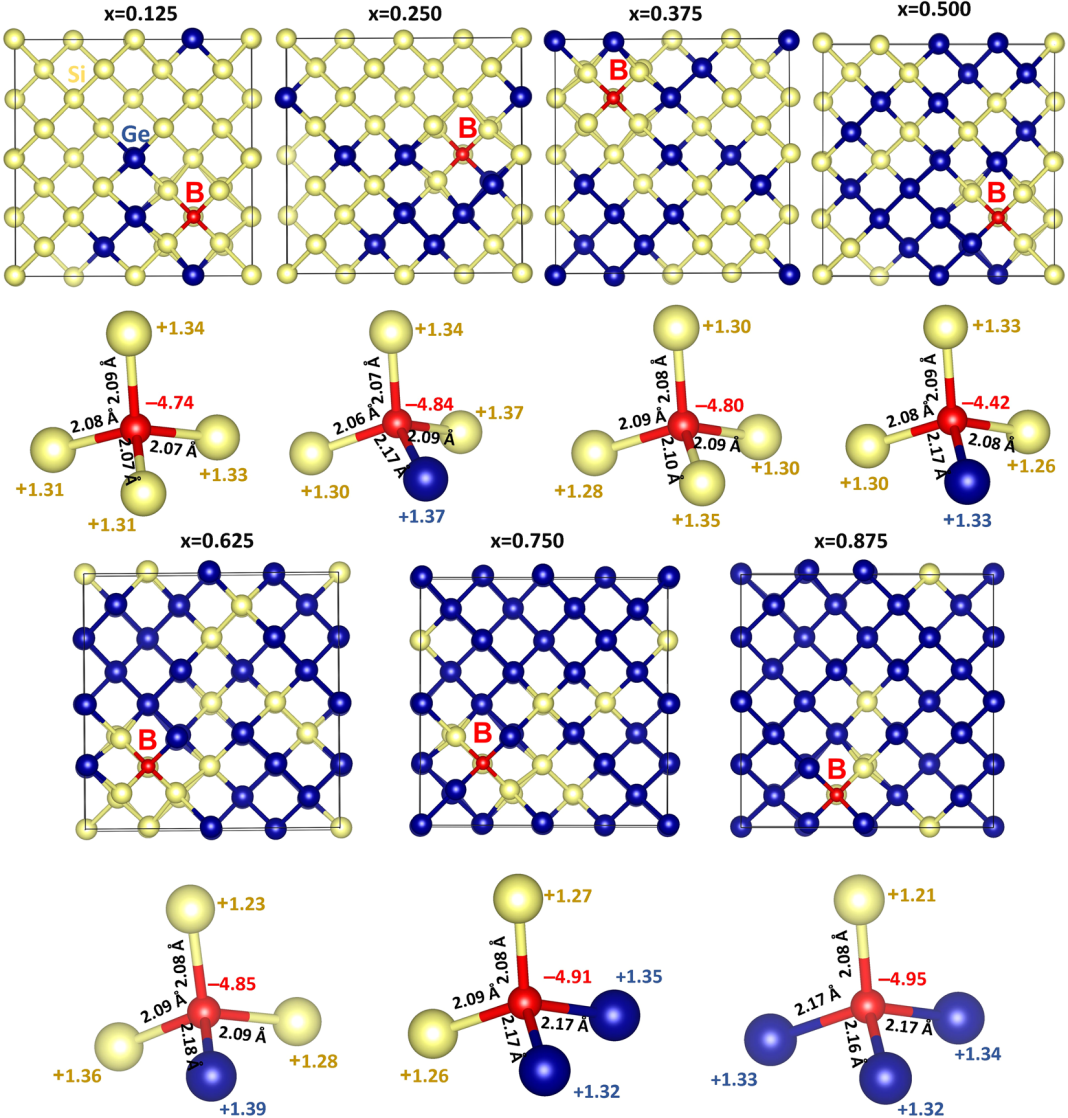
Optimised structures of seven different boron substitutional defect configurations in Si_1−x_Ge_x_ alloys. Bader charges on the B and its nearest neighbour atoms and bond distances (B-Si and B-Ge) are also shown.

**Figure 8:**
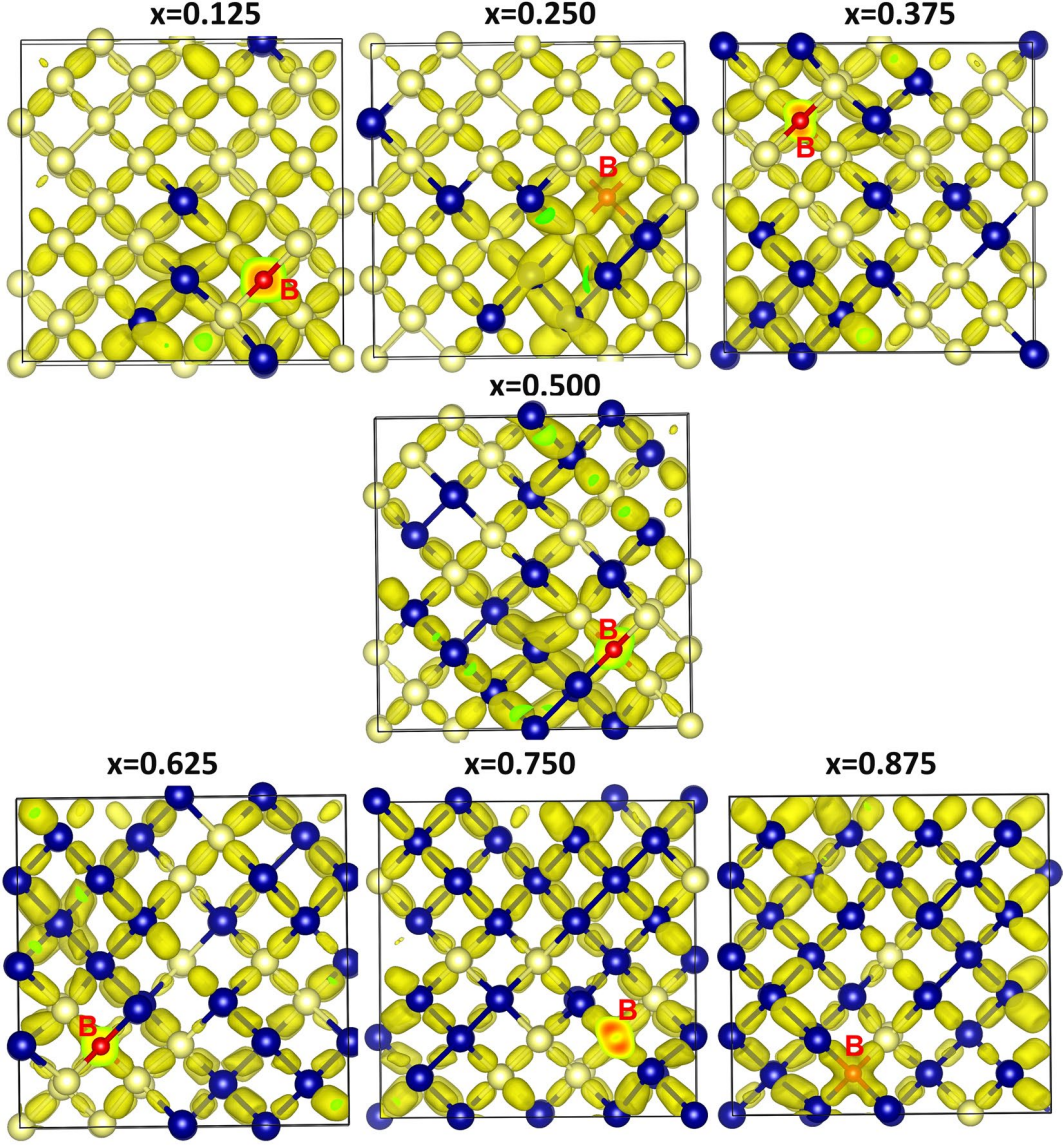
Surface of the constant charge density showing the interaction of boron in each of the seven different configurations in Si_1−x_Ge_x_ alloys.

**Figure 9:**
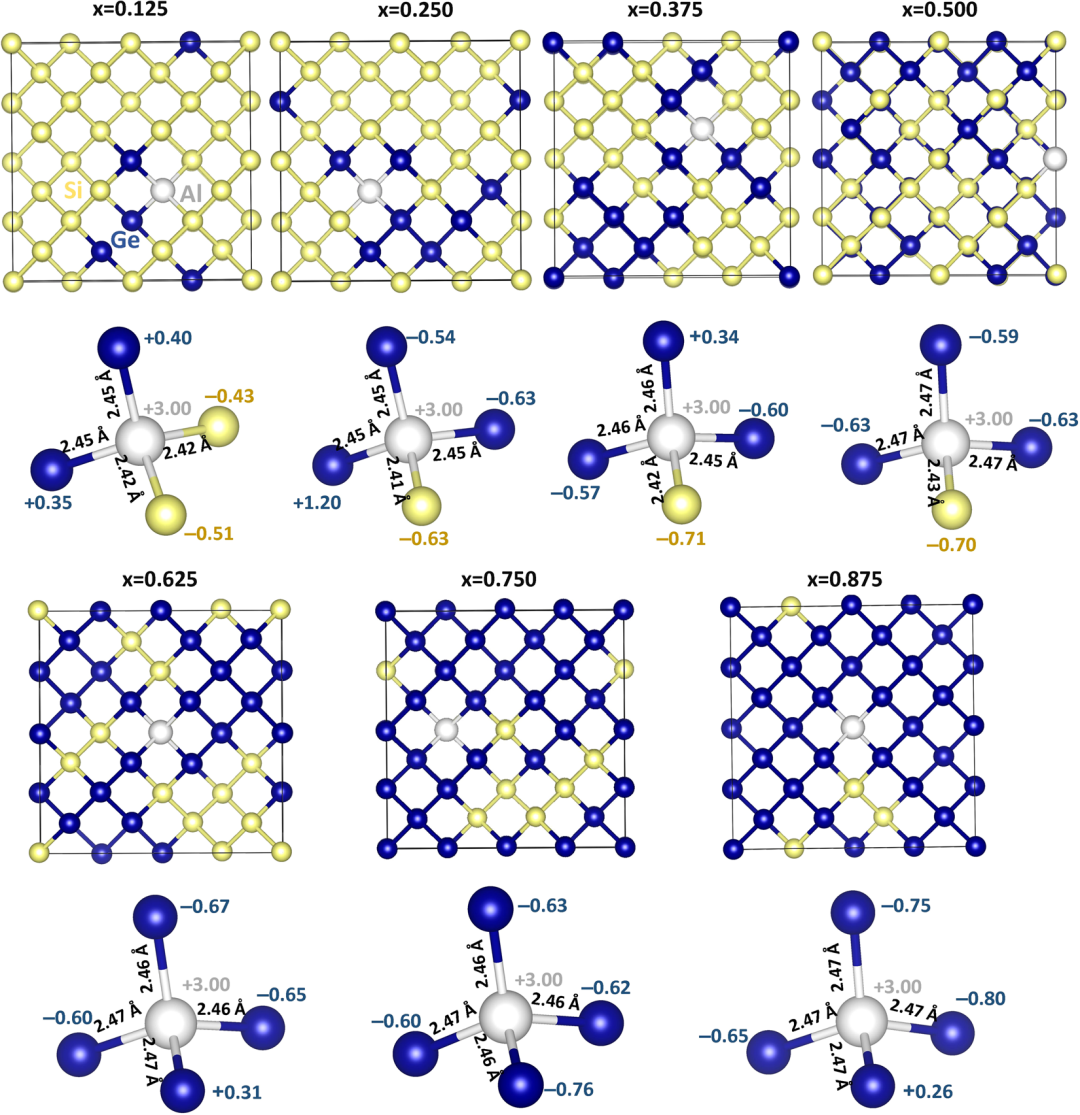
Optimised structures of seven different aluminium substitutional defect configurations in Si_1−x_Ge_x_ alloys. Bader charges on the Al and its nearest neighbour atoms and bond distances (Al-Si and Al-Ge) are also shown.

**Figure 10:**
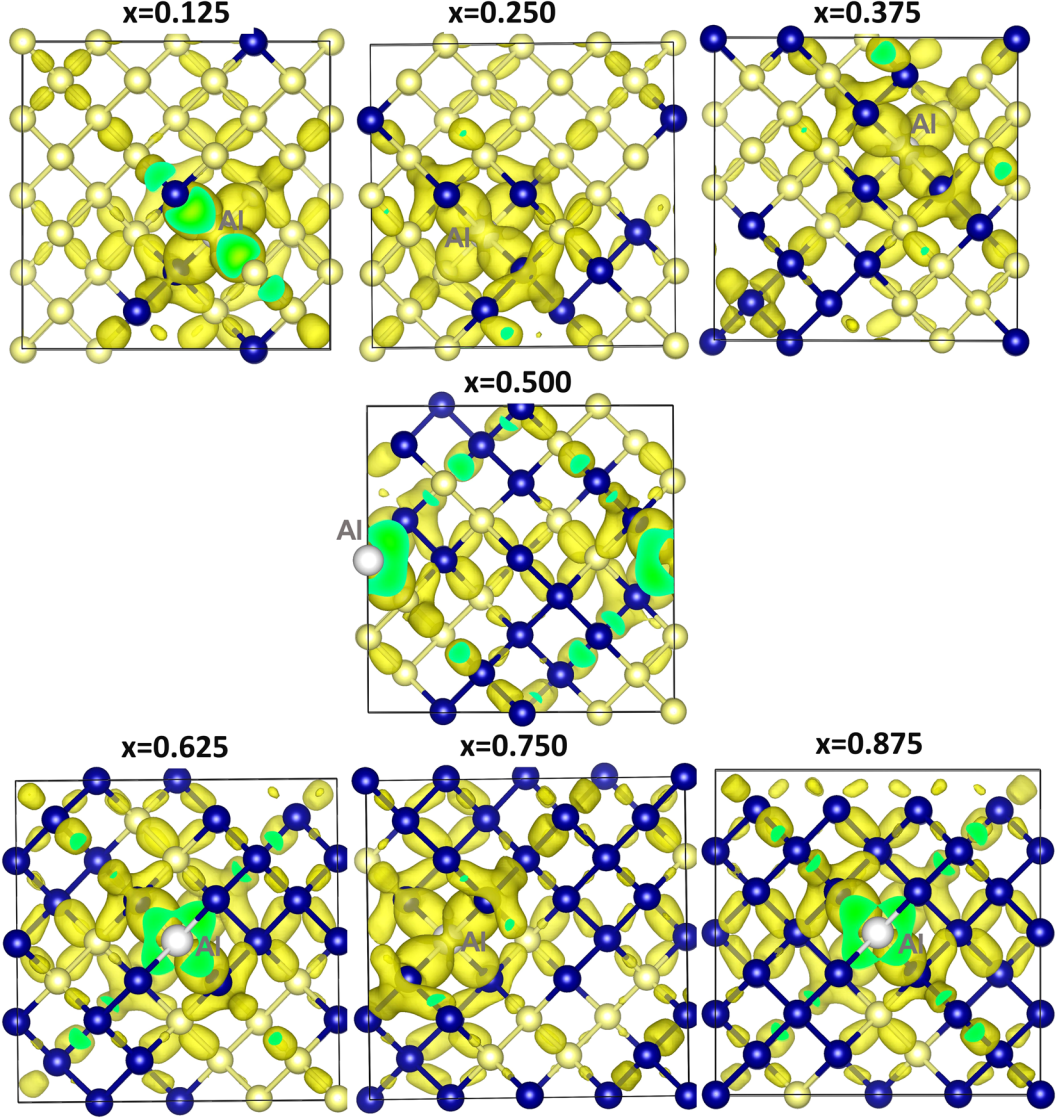
Surface of the constant charge density showing the interaction of aluminium in each of the seven different configurations in Si_1−x_Ge_x_ alloys.

Next we discuss the Bader charges on *p*-type dopants and nearest neighbor atoms by considering the electronegativities and ionization potentials of dopant atoms, Si and Ge. Electronegativity values^36^ reported for B, Al Ga and In are 2.04, 1.61, 1.81 and 1.78. Electronegativity difference between each dopant and Si (or Ge) is calculated and reported in Fig. 5. While positive value is observed for B, other dopants exhibit negative values. Ionization potential difference is positive for B and negative for other dopants (refer to Fig. 5b). Positive values of electronegativity difference and ionization potential difference noted for B reflects in the negative charge (−4.96) on it. Negative values noted for other dopants indicate that they are highly unlikely to gain electron from Si or Ge and highly likely to lose electrons to become positively charged. This is reflected in the positive Bader charge (+3.00) observed on Al, Ga and In. Figure 6 shows the Bader charge on each dopant and total Bader charge on the nearest neighbour atoms. In the case of B, in all seven configurations B atom is negatively charged and the nearest neighbour atoms are positively charged. Optimized structures, bond distances and Bader charges are shown in Fig. 7. Boron forms a tetrahedral coordination with Si and Ge in all configurations. Shorter B-Si distance compared to B-Ge is due to the smaller atomic radius of Si than that of Ge. In Fig. 8, we show the charge density plots of B. Aluminium forms a tetrahedral coordination with the nearest neighbour Si or Ge atoms. Bader charge on Al in each configuration is +3.00. The total charge on the nearest neighbour atoms are negative in all cases but not equal to −3.00. Figure 9 shows the relaxed structures, bond distances and Bader charges of Al and corresponding charge density plots are shown in Fig. 10. The tetrahedral coordination is observed for gallium in Si_1-x_Ge_x_. The Bader charge is +3.00 on Ga. Total Bader charge on the nearest neighbour atoms is negative and the magnitude differs from −1.20 to −2.50 (refer to Figure S7). The remaining negative charges should have been spread out on the second nearest neighbor atoms. Figure S8 exhibits the charge density plots associated with Ga. Finally, the Bader charge on the indium is +3.00 meaning that it completely loses its outer most three electrons. In the first five configurations, total Bader charge of the nearest neighbor atoms is positive meaning that some electrons should have been localized further away from the In. As expected, bond distances are quite longer than that noted for B, Al and Ga (refer to Figure S9). Charge density plots are shown in Figure S10.

### Summary

Electronic structure calculations were employed to study substitutional doping in a range of Si_1-*x*_Ge_*x*_ alloys. It is demonstrated that the Bader charge of the substitutional dopants is dependent upon their nearest neighbours and the composition of the alloy. It is found that *n*-type dopants (N, P, As and Sb) accept electrons from the nearest neighbour atoms (Si and Ge). In particular N, P and As gain almost 3 electrons to form stable X^3‒^ ion. Sb accepts only a small amount of electrons as its electronegativity is very closer to the values of Si and Ge. Among *p*-type dopants, boron accepts electrons while other dopants (Al, Ga and In) loses their outer most three electrons. This is due to the higher electrone gativity of B than that of Si and Ge. The present methodology can be employed to energy materials (fuel cell, battery materials) related systems were random alloys are important^38,39^. This is because in these structurally disordered materials the local environments are bound to influence the dopant diffusion and the electronic properties, which are in turn important for the applicability of the materials in fuel cell and battery materials^38,39^.

### Methods

All the calculations were spin polarized using the VASP DFT code that uses plane wave basis sets^40,41^. Exchange correlation was modeled using generalized gradient approximation (GGA) as parameterized by Perdew, Burke and Ernzerhof^42^. All the calculations were performed on 64-atomic site supercell consisting of two 32-atomic site SQS cells, a plane wave basis set, cut-off energy of 500 eV and a 4 × 4 × 4 Monkhorst-Pack^43^ *k*-point mesh (36 *k* points). Constant pressure conditions (atomic position and simulation box were relaxed) implanted via a conjugate gradient algorithm^44^. Convergence criteria dictated that forces on the atoms and stress tensors were less than 0.001 eV/Å and 0.002 GPa respectively. Semi-empirical dispersion was introduced in the simulations^45^. Bader charge analysis^32,33^ was employed to calculate the charges on the substitutional atom and its nearest neighbor atoms. The 32-atomic site SQS cells of Si_1−x_Ge_x_ (x = 0.875, 0.750, 0.625, 0.500, 0.375, 0.250, 0.125) used here were previously reported^12,25^.

## Supplementary information

Supplementary information.
