# Supplementary material for: Electronegativity and doping in Si1-xGex alloys
Source: Sci Rep. 2020 May 4;10:7459. doi: 10.1038/s41598-020-64403-8 (PMC7198609; doi:10.1038/s41598-020-64403-8)
Supplement: Supplementary file 1 — Supplementary information. [file 41598_2020_64403_MOESM1_ESM.docx]

Supporting Information

**Electronegativity and doping in Si_1-_*_x_*Ge*_x_* alloys**

# Stavros-Richard G. Christopoulos^1^, Navaratnarajah Kuganathan^1,2^ & Alexander Chroneos,^1,2,b)*^

*^1^Faculty of Engineering, Environment and Computing, Coventry University, Priory Street, Coventry CV1 5FB, United Kingdom*

*^2^Department of Materials, Imperial College London, London SW7 2AZ, United Kingdom*

**Table S1.** Calculated bond distances [Y-Si or Y-Ge (Y= B, Al, Ga and In)] in the optimised structures of *n*-type dopants interacting Si_1-x_Ge_x_ alloys

| **B•Si_1-x_Ge_x_** | **Bond distance (Å)** | |
| --- | --- | --- |
|  | **B‒Si** | **B‒Ge** |
| x=0.125 | 2.08 | ‒ |
| x=0.250 | 2.07 | 2.17 |
| x=0.375 | 2.09 | 2.17 |
| x=0.500 | 2.08 |  |
| x=0.625 | 2.09 | 2.18 |
| x=0.750 | 2.09 | 2.17 |
| x=0.875 | 2.08 | 2.17 |
|  | | |
| **Al•Si_1-x_Ge_x_** | **Al‒Si** | **Al‒Ge** |
| x=0.125 | 2.42 | 2.45 |
| x=0.250 | 2.41 | 2.45 |
| x=0.375 | 2.42 | 2.45 |
| x=0.500 | 2.43 | 2.47 |
| x=0.625 | ‒ | 2.47 |
| x=0.750 | ‒ | 2.46 |
| x=0.875 | ‒ | 2.47 |
|  | | |
| **Ga•Si_1-x_Ge_x_** | **Ga‒Si** | **Ga‒Ge** |
| x=0.125 | 2.40 | 2.44 |
| x=0.250 | 2.39 | 2.44 |
| x=0.375 | 2.41 | 2.45 |
| x=0.500 | 2.41 | 2.45 |
| x=0.625 | ‒ | 2.45 |
| x=0.750 | ‒ | 2.45 |
| x=0.875 | ‒ | 2.46 |
|  | | |
| **In•Si_1-x_Ge_x_** | **In‒Si** | **In‒Ge** |
| x=0.125 | 2.55 | 2.58 |
| x=0.250 | 2.55 | 2.59 |
| x=0.375 | 2.55 | 2.59 |
| x=0.500 | 2.55 | 2.59 |
| x=0.625 | ‒ | 2.59 |
| x=0.750 | ‒ | 2.59 |
| x=0.875 | ‒ | 2.59 |

**Table S2.** Calculated bond distances [Y-Si or X-Ge (Y= N, P, As and Sb)] in the optimised structures of *p*-type dopants interacting Si_1-x_Ge_x_ alloys

| **N•Si_1-x_Ge_x_** | **Bond distance (Å)** | |
| --- | --- | --- |
|  | **N‒Si** | **N‒Ge** |
| x=0.125 | 1.86 | ‒ |
| x=0.250 | 1.85 | ‒ |
| x=0.375 | 1.85 | ‒ |
| x=0.500 | 1.85 | ‒ |
| x=0.625 | 1.86 | ‒ |
| x=0.750 | 1.82 | 2.09 |
| x=0.875 | 1.90 | 2.21 |
|  | | |
| **P•Si_1-x_Ge_x_** | **P‒Si** | **P‒Ge** |
| x=0.125 | 2.35 | 2.45 |
| x=0.250 | 2.35 | 2.46 |
| x=0.375 | 2.35 | 2.45 |
| x=0.500 | 2.36 | 2.46 |
| x=0.625 | 2.35 | 2.44 |
| x=0.750 | ‒ | 2.45 |
| x=0.875 | 2.35 | 2.47 |
|  | | |
| **As•Si_1-x_Ge_x_** | **As‒Si** | **As‒Ge** |
| x=0.125 | 2.44 | 2.52 |
| x=0.250 | 2.44 | 2.53 |
| x=0.375 | 2.44 | 2.52 |
| x=0.500 | 2.44 | 2.53 |
| x=0.625 | 2.44 | 2.53 |
| x=0.750 | ‒ | 2.53 |
| x=0.875 | ‒ | 2.53 |
|  | | |
| **Sb•Si_1-x_Ge_x_** | **Sb‒Si** | **Sb‒Ge** |
| x=0.125 | 2.58 | 2.64 |
| x=0.250 | 2.58 | 2.64 |
| x=0.375 | 2.58 | 2.65 |
| x=0.500 | 2.59 | 2.65 |
| x=0.625 | ‒ | 2.65 |
| x=0.750 | ‒ | 2.66 |
| x=0.875 | 2.61 | 2.67 |


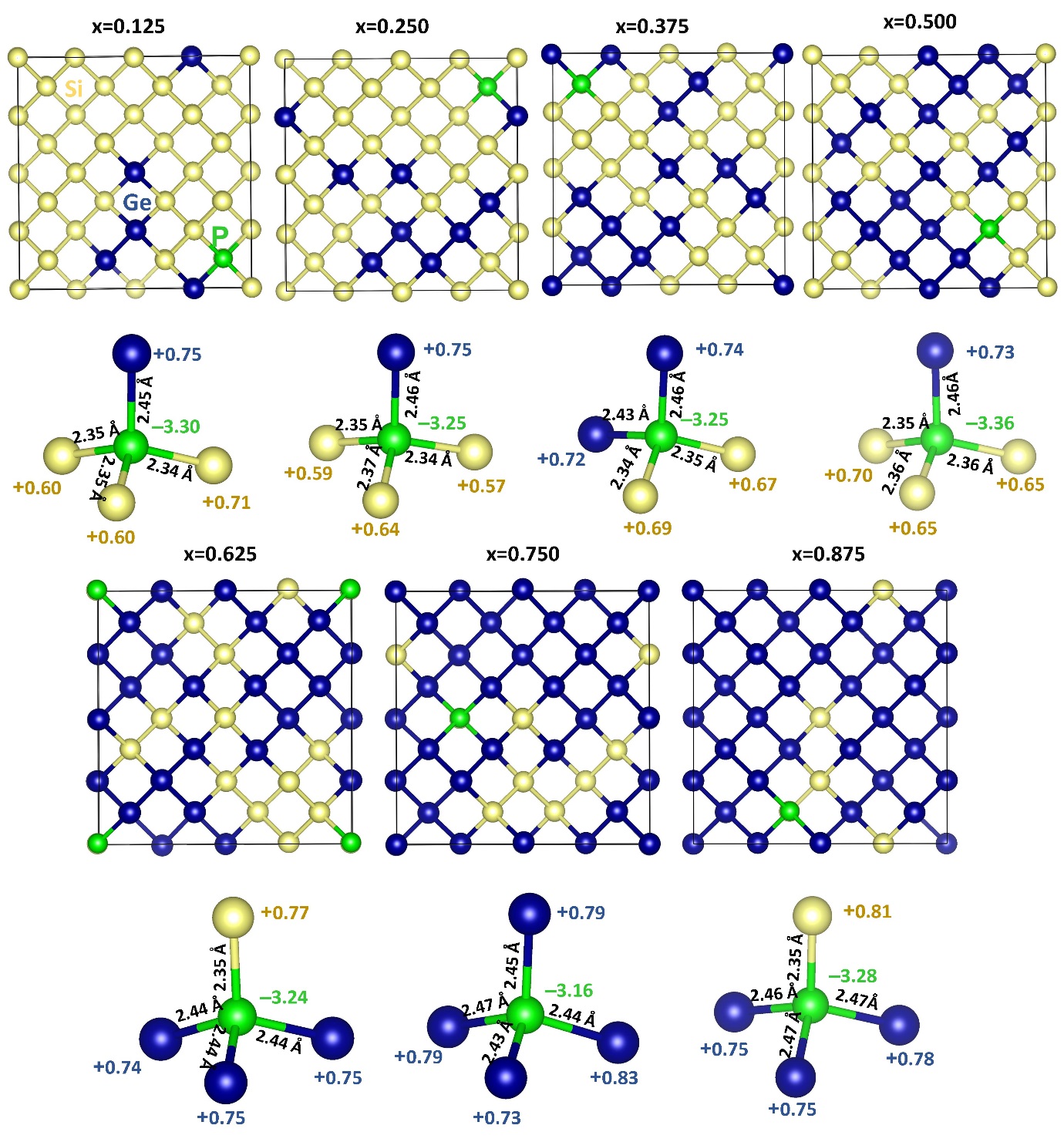


**Figure S1.** Optimised structures of seven different configurations of phosphorous interacting Si_1-x_Ge_x_ alloys. Bader charge on the P and its nearest neighbour atoms and bond distances (P-Si and P-Ge) are also shown.


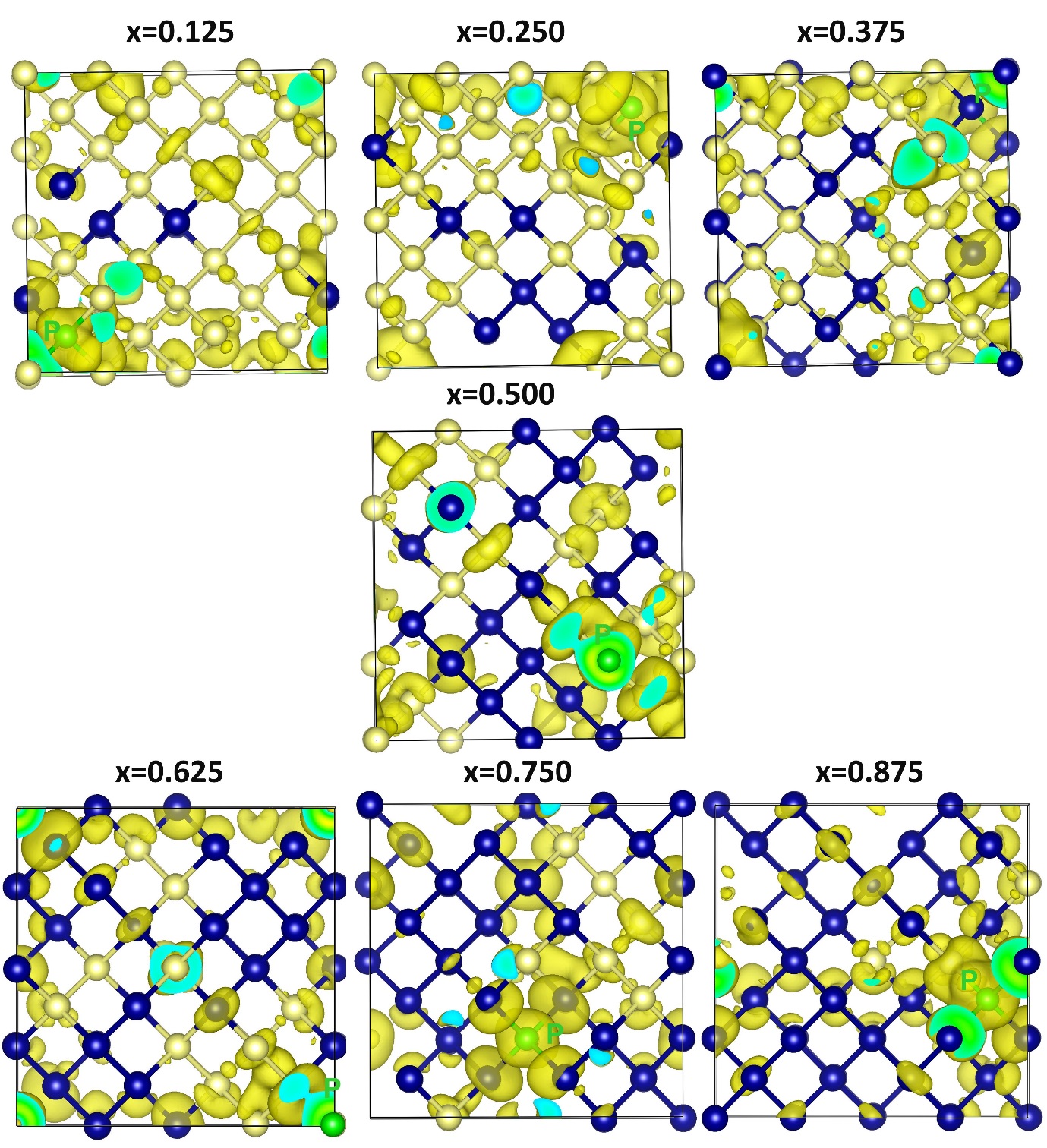


**Figure S2.** Surface of the constant charge density showing the interaction of phosphorous with Si_1-x_Ge_x_ alloys.


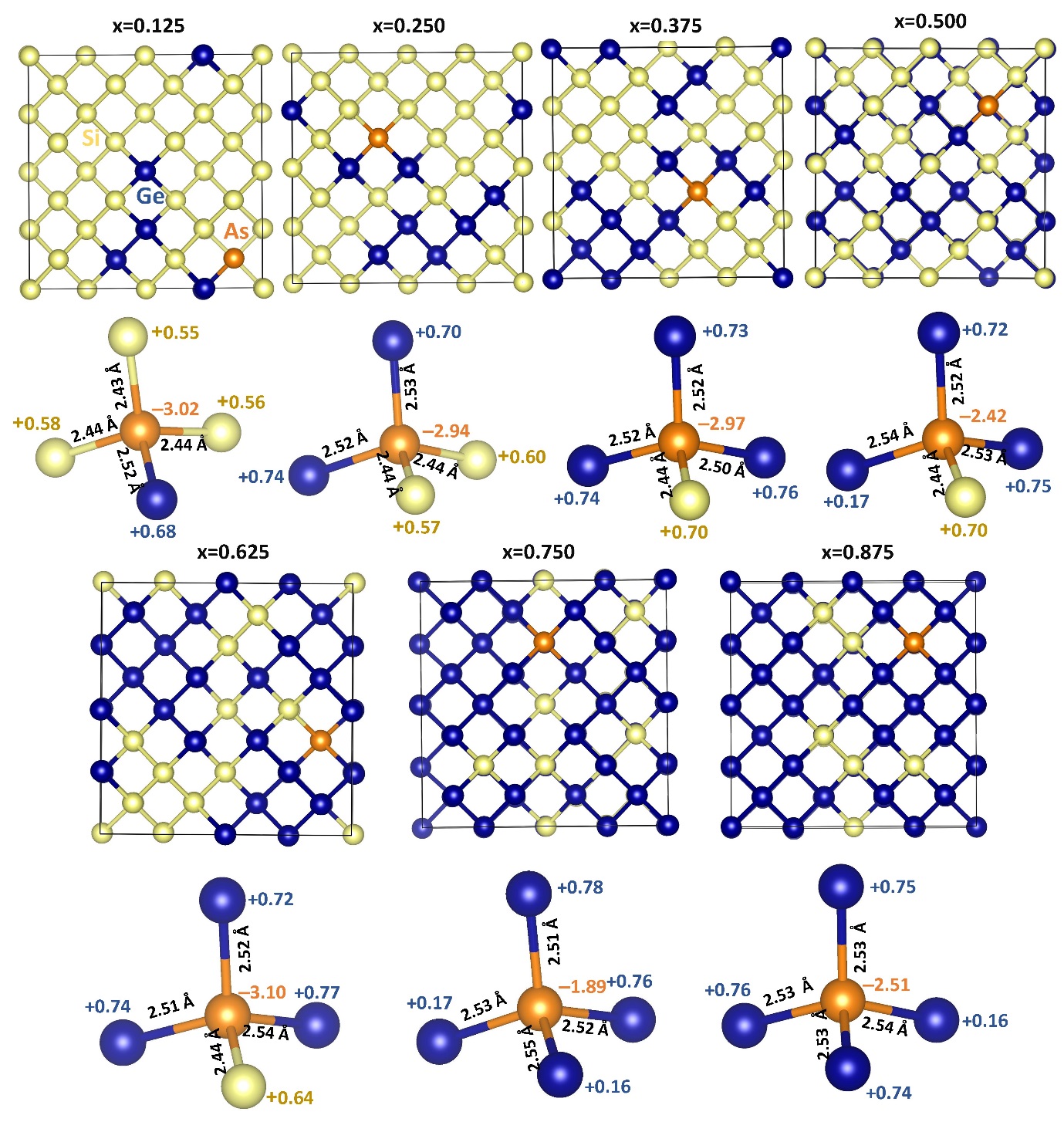


**Figure S3.** Optimised structures of seven different configurations of arsenic interacting Si_1-x_Ge_x_ alloys. Bader charge on the As and its nearest neighbour atoms and bond distances (As-Si and As-Ge) are also shown.


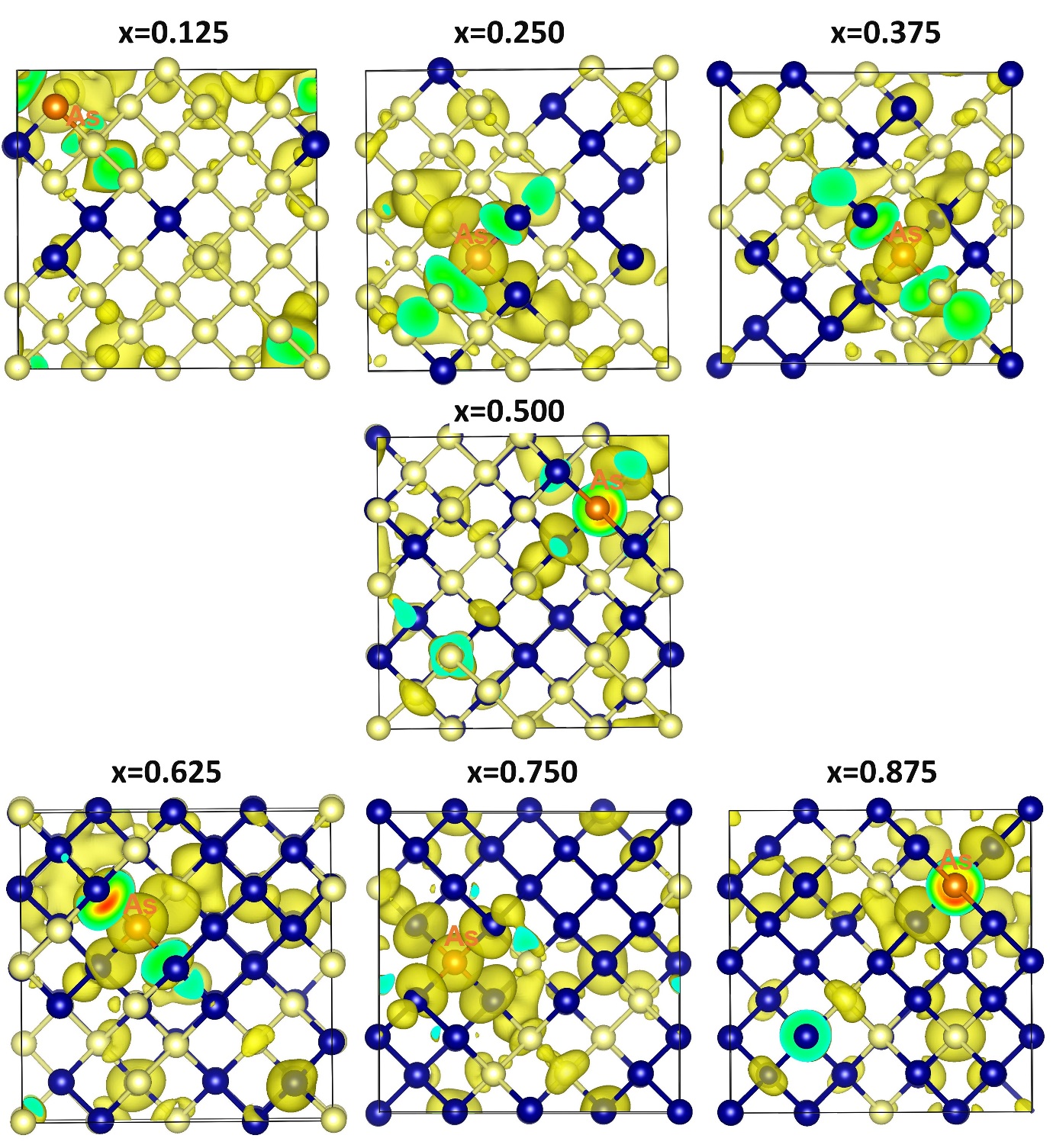


**Figure S4.** Surface of the constant charge density showing the interaction of arsenic with Si_1-x_Ge_x_ alloys.


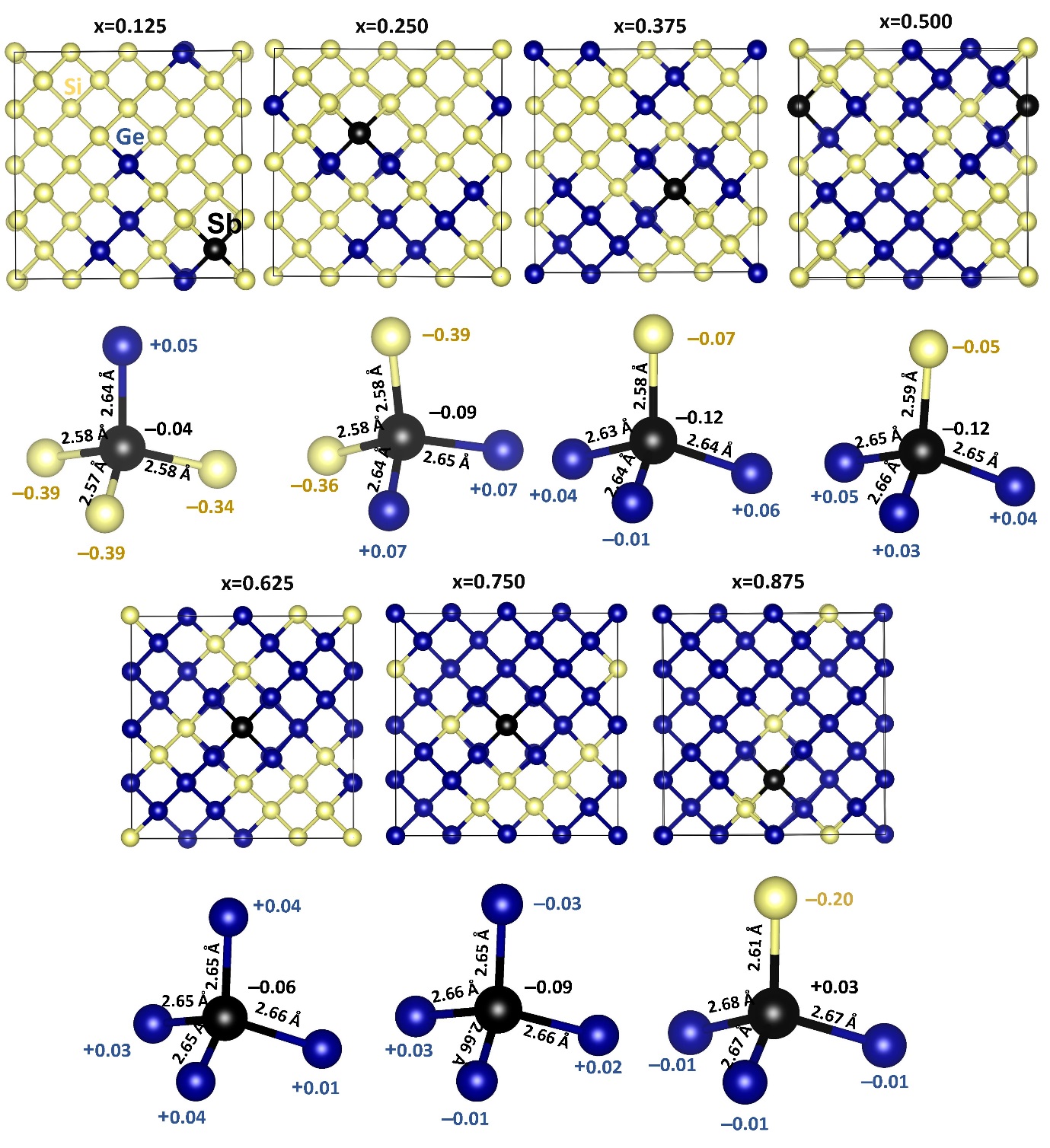


**Figure S5.** Optimised structures of seven different configurations of antimony interacting Si_1-x_Ge_x_ alloys. Bader charge on the Sb and its nearest neighbour atoms and bond distances (Sb-Si and Sb-Ge) are also shown.


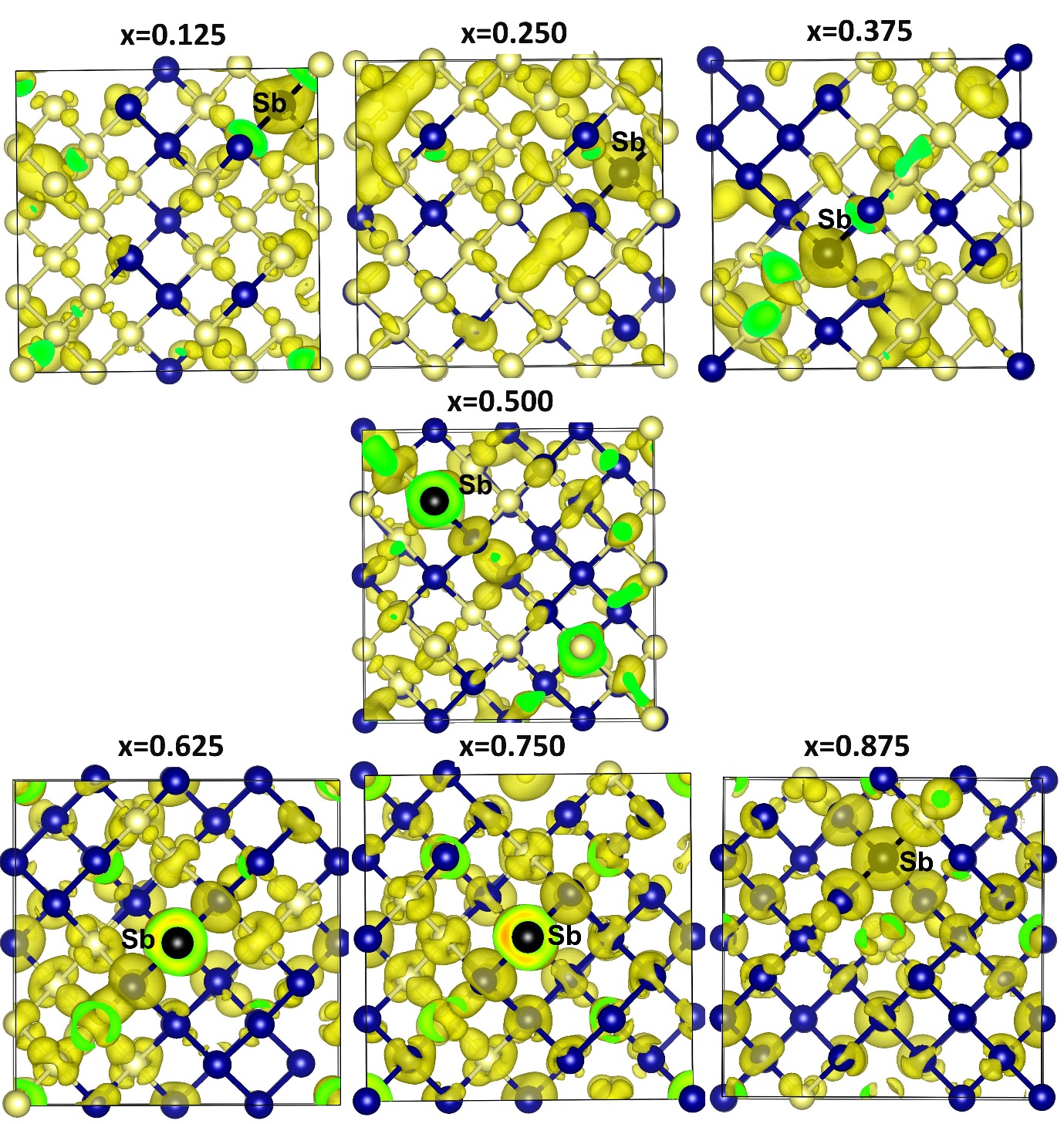


**Figure S6.** Surface of the constant charge density showing the interaction of antimony with Si_1-x_Ge_x_ alloys.


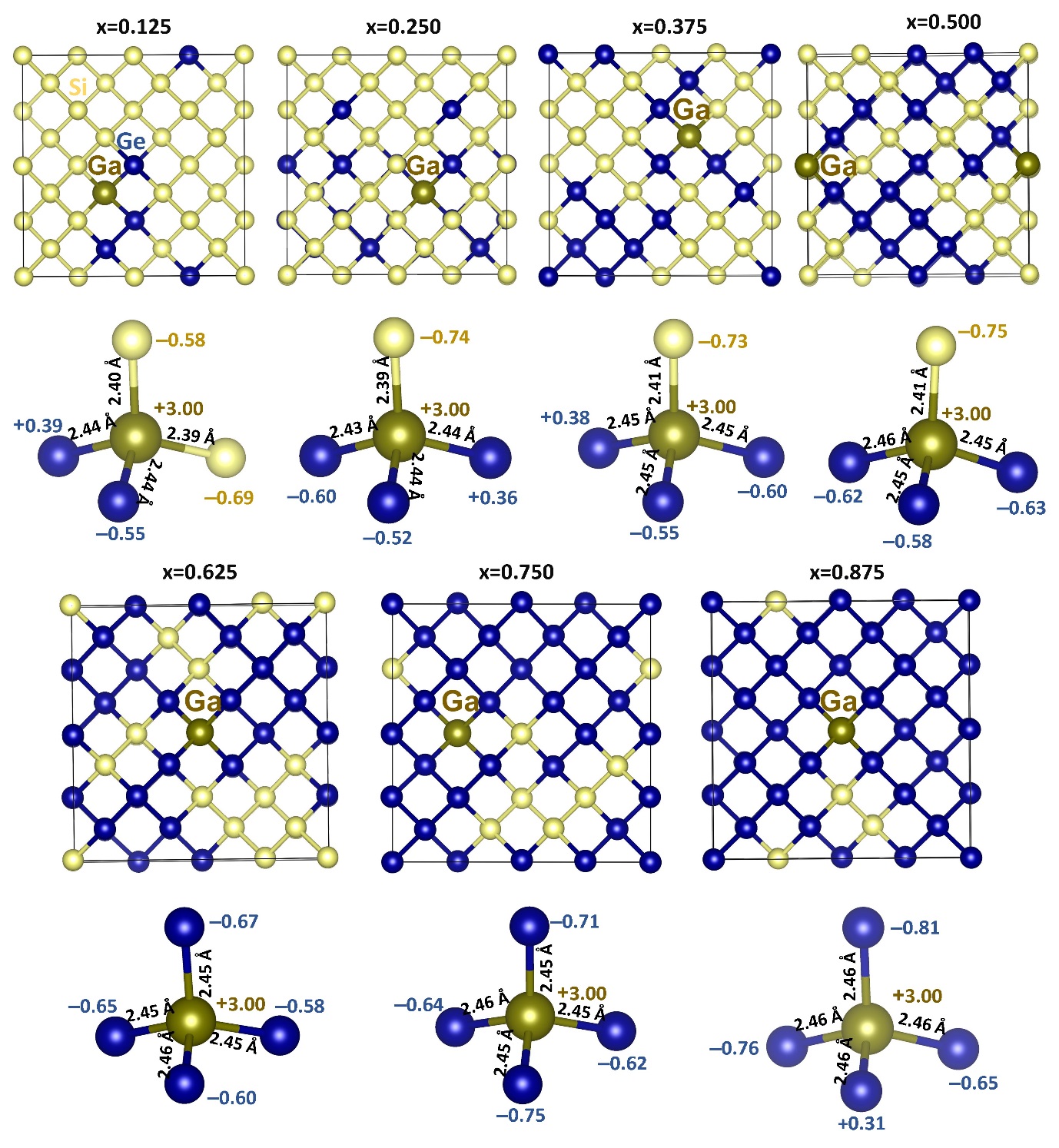


**Figure S7.** Optimised structures of seven different configurations of gallium interacting Si_1-x_Ge_x_ alloys. Bader charge on the Ga and its nearest neighbour atoms and bond distances (Ga-Si and Ga-Ge) are also shown.


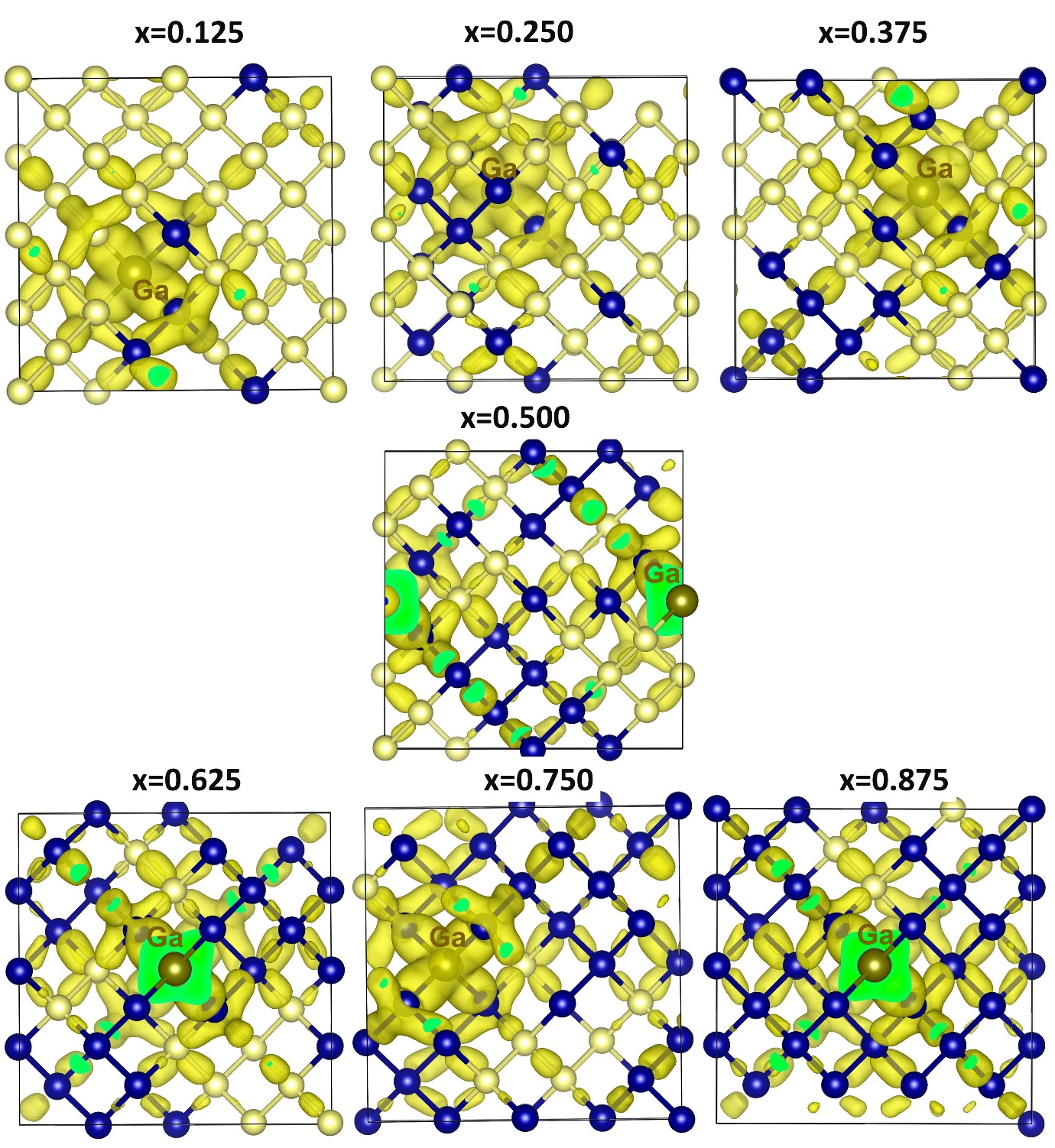


**Figure S8.** Surface of the constant charge density showing the interaction of gallium with Si_1_-_x_Ge_x_ alloys.


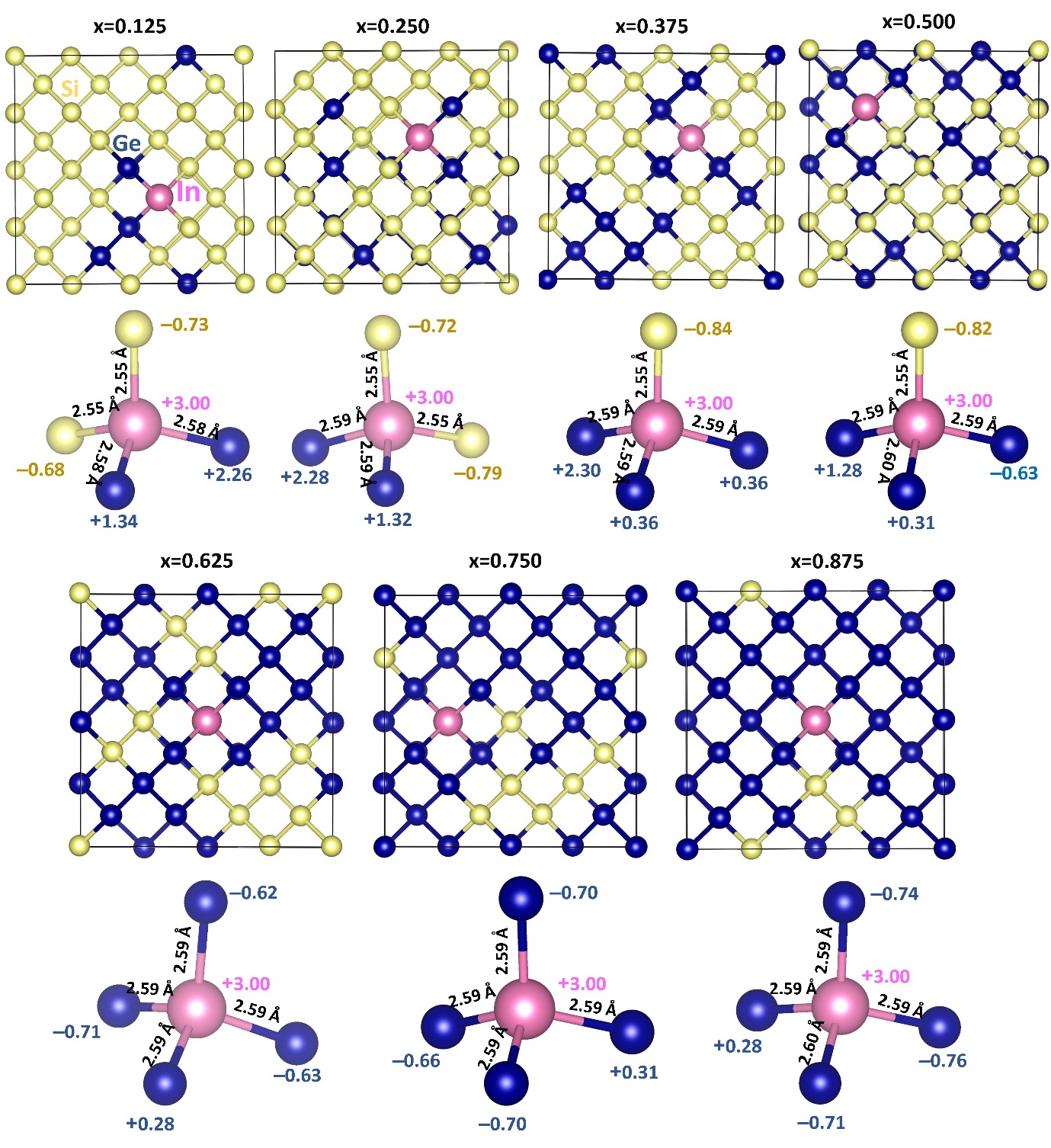


**Figure S9.** Optimised structures of seven different configurations of indium interacting Si_1-x_Ge_x_ alloys. Bader charge on the In and its nearest neighbour atoms and bond distances (In-Si and In-Ge) are also shown.


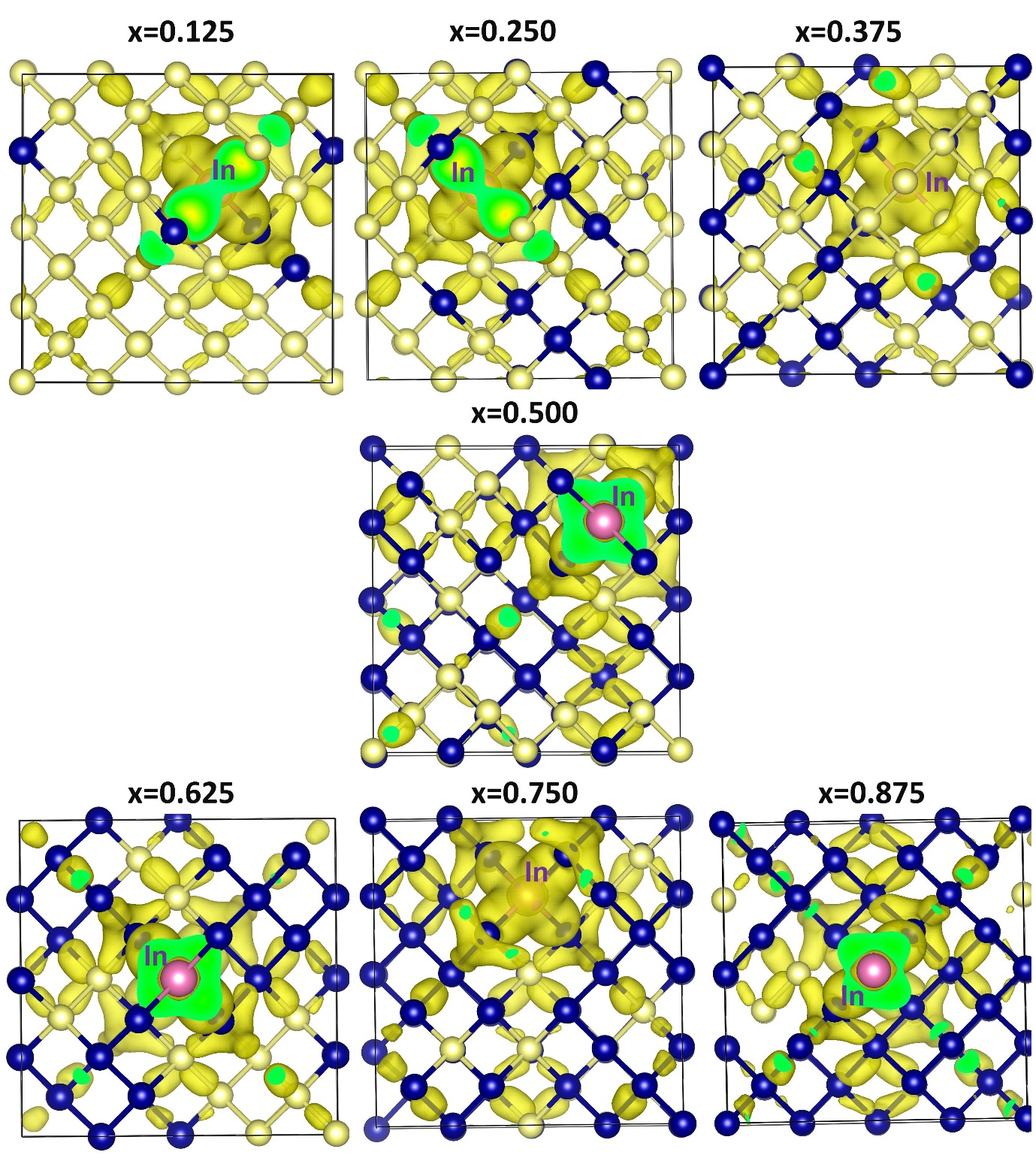


**Figure S10.** Surface of the constant charge density showing the interaction of indium with Si_1-x_Ge_x_ alloys.
